# Spin-charge-lattice coupling in YBaCuFeO_5_: Optical properties and first-principles calculations

**DOI:** 10.1038/s41598-019-39031-6

**Published:** 2019-03-01

**Authors:** H. W. Chen, Y.-W. Chen, J.-L. Kuo, Y. C. Lai, F. C. Chou, C. H. Du, H. L. Liu

**Affiliations:** 1 Department of Physics, National Taiwan Normal University, Taipei, 11677 Taiwan; 2 Institute of Atomic and Molecular Sciences, Academia Sinica, Taipei, 10617 Taiwan; 3 Center for Condensed Matter Sciences, National Taiwan University, Taipei, 10617 Taiwan; 4 Department of Physics, Tamkang University, Tamsui, New Taipei City 25137 Taiwan

## Abstract

We combined spectroscopic ellipsometry, Raman scattering spectroscopy, and first-principles calculations to explore the optical properties of YBaCuFeO_5_ single crystals. Measuring the optical absorption spectrum of YBaCuFeO_5_ at room temperature revealed a direct optical band gap at approximately 1.41 eV and five bands near 1.69, 2.47, 3.16, 4.26, and 5.54 eV. Based on first-principles calculations, the observed optical excitations were appropriately assigned. Analysis of the temperature dependence of the band gap indicated anomalies in antiferromagnetic phase transition at 455 and 175 K. Additionally, a hardening in the frequency of the *E*_*g*_ phonon mode was observed at 175 K. The value of the spin–phonon coupling constant was 15.7 mRy/Å^2^. These results suggest a complex nature of spin–charge–lattice interactions in YBaCuFeO_5_.

## Introduction

Since YBaCuFeO_5_ was synthesized in 1988^1^ and treated as an impurity phase in the Fe-doped high-temperature copper oxides of YBa_2_Cu_3_O_7_, this compound has received substantial research attention because of its complex physical properties and potential practical applications^2–14^. YBaCuFeO_5_ is tetragonal in structure and belongs to the P4/mmm space group^1,4,6,7^. The building block consists of a CuFeO_10_ bilayer of corner-sharing CuO_5_ and FeO_5_ square pyramids. Y^3+^ cations separate the CuFeO_10_ bilayer, and the Ba^2+^ ions are located within the bilayer; this structure resembles the crystal structure in YBa_2_Cu_3_O_7_. Much of the research interest concerning YBaCuFeO_5_ has been focused on the compound’s complex magnetic structures^2,4–9^. YBaCuFeO_5_ displays a commensurate antiferromagnetic structure at approximately T_N1_ = 440 K and an incommensurate spiral antiferromagnetic structure at temperatures below T_N2_ = 230 K. Recent reports of magnetism-driven ferroelectricity at T_N2_ = 230 K and a large electric polarization (0.4 μC/cm^2^) observed in YBaCuFeO_5_^10,11,13^ have led to further explorations of the possibilities for new functional devices based on the magnetoelectric effect of this material^15–18^.

Numerous studies have examined magnetization, Mossbauer, neutron powder diffraction, and dielectric measurements, but the optical and phonon properties of YBaCuFeO_5_ have remained largely unexplored^3^. Moreover, previous studies on YBaCuFeO_5_ have been limited to powder samples. Recently, we modified the traveling-solvent floating-zone technique and used it to synthesize high-quality YBaCuFeO_5_ single crystals^19^. Consecutive anisotropic antiferromagnetic phase transitions were observed at approximately 455 and 170 K^19,20^. In the present study, we combined spectroscopic ellipsometry, Raman scattering spectroscopy, and first-principles calculations to determine the electronic structure and lattice dynamics of YBaCuFeO_5_. Clarification of the microscopic characteristics that govern the material’s optical and phononic excitations is critical for further development of device applications. In this study, we also examined the correlation of the optical response with the magnetic phase transitions of YBaCuFeO_5_. The findings of this investigation may help elucidate the nature of spin–charge–lattice coupling in this system and in related multiferroic materials.

## Technical Details

### Experiment

**Figure 1:**
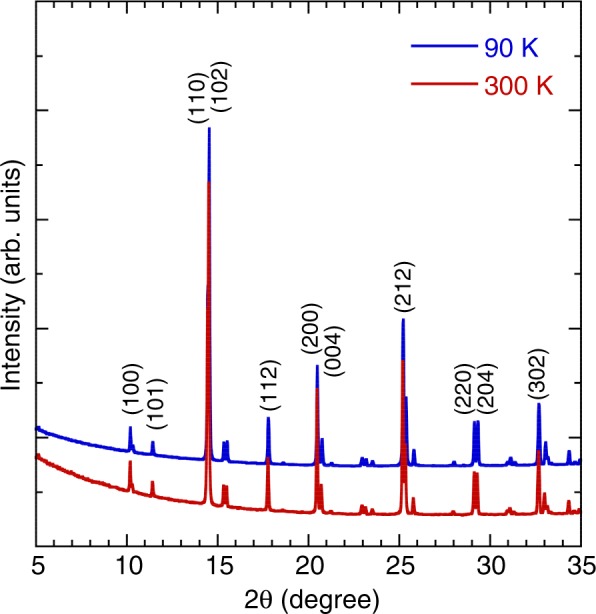
X-ray powder diffraction patterns of YBaCuFeO_5_ at 300 and 90 K.

YBaCuFeO_5_ single crystals were grown using a modified traveling-solvent floating-zone method. The details of sample preparation were as reported previously^19^. The crystals that formed on the *bc*-surface exhibited disk shapes of 5-mm diameter and 2-mm thickness. The (100) axis is the floating-zone growth direction. For each batch, crystals were characterized using synchrotron x-ray powder diffraction, dc resistivity, and magnetization measurements^19,20^. Figure 1 presents the profiles for x-ray powder diffraction of YBaCuFeO_5_ at 300 and 90 K. The spectra did not change significantly with temperature, which implied that no structural phase transition occurred within the temperature range investigated. Only after cooling below 300 K did YBaCuFeO_5_ exhibit a decrease in the *a* and *c* lattice constants.

Spectroscopic ellipsometric measurements were conducted under angles of incidence between 60° and 75° and over a spectral range of 0.73 to 6.42 eV using a Woollam M-2000U ellipsometer. For temperature-dependent measurements between 4.3 and 500 K, the ellipsometer was equipped with an ultrahigh-vacuum cryostat. Raman scattering experiments were performed in a backscattering geometry with a laser-excitation wavelength of 532 nm. The linearly polarized light was focused to a 3-*μ*m-diameter spot on the sample surface, and the scattered light was collected and dispersed using a SENTERRA spectrometer equipped with a 1024-pixel-wide charge-coupled detector. The polarizations of incident and scattered light were set such that the incident and scattered lights were parallel and perpendicular (cross) to each other. The spectral resolutions achieved using these instruments are typically less than 1 cm^−1^. To avoid heating effects, the laser power was set to 0.2 mW. The sample was placed in a continuous-flow helium cryostat and Linkam heating stage, enabling measurements of the sample under temperatures of 10–700 K.

### Theoretical methods

To investigate the electronic structure of YBaCuFeO_5_, we modeled several YBaCuFeO_5_ systems using density functional theory (DFT) calculations in the Vienna ab initio Simulation Package (VASP)^21,22^. The electron wavefunctions were expanded in plane wave basis sets with an energy cutoff of 550 eV. Perdew–Burke–Ernzerhof (PBE) functional approximation was applied to the electron exchange correlation energy^23^. The 5*s*4*p*4*d*4*s*, 6*s*5*s*5*p*, 4*s*3*d*, 4*s*3*d*, and 2*s*2*p* orbitals of Y, Ba, Cu, Fe, and O atom species were treated as valence electrons, and the pseudopotentials of core electrons were generated using the projector augmented-wave method^24,25^. A 5 × 5 × 3 gamma-centered k-point mesh was applied to establish the optimized structures and absorption coefficients. The bandgap sizes of metal oxides are underestimated in DFT calculations; therefore, additional Hubbard-U potentials^26^ were applied to Cu (U = 1 eV, J = 0 eV) and Fe (U = 6 eV, J = 0 eV) atom species. To enable observation of the G-type antiferromagnetic feature and of variation in the arrangement of Cu and Fe atoms, models with supercells comprising 2 × 2 × 2 formula cells were constructed (see supplementary information). In terms of one formula cell, the optimized lattice constants, *a* and *c*, ranged from 3.884 to 3.917 Å and 7.786 to 7.847 Å; these values were in favorable agreement with the experimental data^20^.

## Results and Discussion

### Electronic excitations

**Figure 2:**
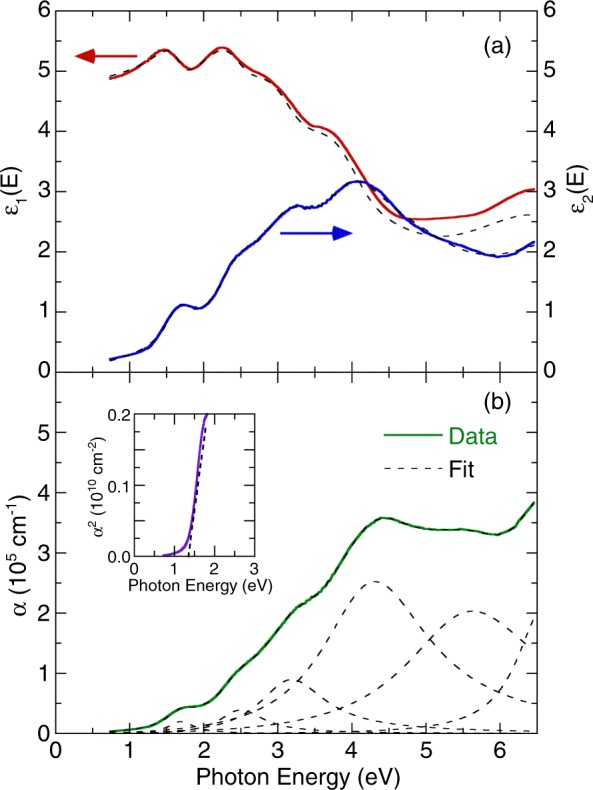
(**a**) Dielectric function for YBaCuFeO_5_ at room temperature. (**b**) Optical absorption coefficient of YBaCuFeO_5_ at room temperature. The dashed lines indicate the best fit from the Lorentzian model. The inset illustrates the direct band gap analysis of YBaCuFeO_5_.

Figure 2(a) presents the room-temperature real *ε*_1_ and imaginary *ε*_2_ parts of the dielectric function *ε*(*ω*) of YBaCuFeO_5_ obtained from spectroscopic ellipsometry analysis. We observed a dispersive response in *ε*_1_ with an overall positive value, which reflected typical behavior for a semiconductor. Optical transitions were identifiable in the spectra based on resonance and antiresonance features, which appeared at the same energy in *ε*_2_ and *ε*_1_, respectively. Notably, the spectrum *ε*_2_ of YBaCuFeO_5_ was dominated by optical transitions, for which detailed analysis is presented subsequently. We fitted the spectra reasonably well using a classical Lorentzian model for the complex dielectric function^27^

1 $${\rm{\varepsilon }}(\omega )={{\rm{\varepsilon }}}_{\infty }+{\sum }_{j=1}^{N}\frac{{\omega }_{pj}^{2}}{({\omega }_{j}^{2}-{\omega }^{2})-i\omega {\gamma }_{j}}$$

where *ω*_j_, *γ*_j_, and *ω*_*pj*_ represent the frequency, damping, and oscillator strength of the *j*th Lorentzian contribution. *ε*_*∞*_ is the permittivity due to high-frequency electronic excitations. Figure 2(b) illustrates the room-temperature optical absorption spectrum. This absorption spectrum can also be modeled reasonably well by using Lorentzian oscillators. As the photon energies increased, the absorption gradually increased; three bands were manifested near 1.69, 2.47, and 3.16 eV; a maximum value was reached at approximately 4.26 eV, and a higher band was manifested at approximately 5.54 eV. In a normal solid, the absorption coefficient *α*(*E*) is accepted and is based on contributions from both direct and indirect band gap transitions^28^. The absorption coefficient is given by

2 $$\alpha (E)=A{(E-{E}_{g,dir})}^{0.5}+B{(E-{E}_{g,indir}\mp {E}_{ph})}^{2},$$

where *E*_*g,dir*_ and *E*_*g,ind*_ are the magnitudes of direct and indirect gaps, respectively; *E*_*ph*_ is the emitted (absorbed) phonon energy, and *A* and *B* are constants. This model, which assumes a simple band shape, enables extraction of the direct energy gap when *α*^2^ is plotted as a function of photon energy. The inset of Fig. 2(b) illustrates the direct band gap of 1.41 ± 0.01 eV at 300 K.

**Figure 3:**
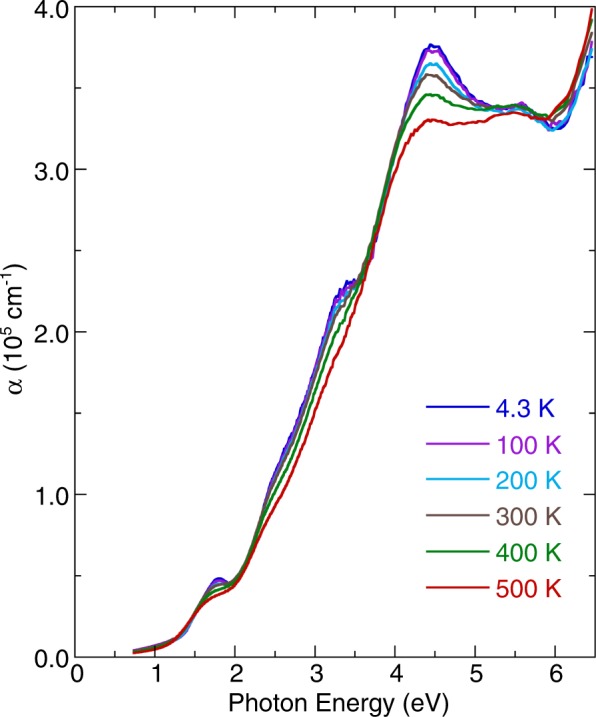
Temperature dependence of the optical absorption spectra of YBaCuFeO_5_.

**Figure 4:**
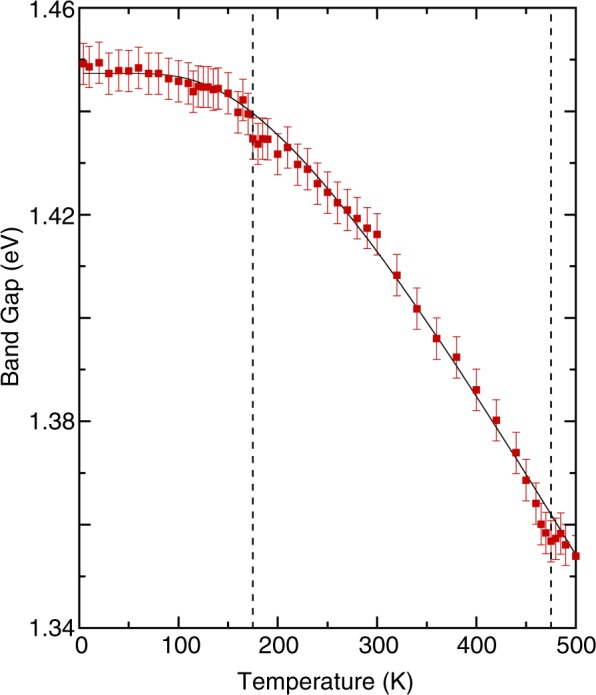
The energy band gap as a function of temperature for YBaCuFeO_5_. The thin solid line indicates the result of the fitting using the Bose–Einstein model. Vertical dashed lines denote transition temperatures.

Figure 3 illustrates the temperature dependence of the optical absorption spectra. As the temperature was lowered, all optical absorptions exhibited shifts of the peak positions to higher energies and narrowing of linewidths. Figure 4 indicates the temperature dependence of the energy band gap. The gap became hardening as the temperature decreased. In principle, the observed blueshift value of the band gap energy with decreasing temperature in semiconductors can be described using the Bose–Einstein model^29^

3 $$\,{E}_{g}(T)={E}_{g}(0)-\frac{2{a}_{B}}{\exp ({{\rm{\Theta }}}_{B}/T)-1},$$

where *E*_*g*_*(0)* is the band gap energy at 0 K; *a*_*B*_ represents the strength of the electron–phonon interactions; and *Θ*_*B*_ is the average phonon temperature. Our fitting results indicated that the band gap energy toward 0 K was approximately 1.45 ± 0.01 eV. The strength of the electron–phonon interactions *a*_*B*_ and the average phonon temperature *Θ*_*B*_ were 102 meV and 496 K, respectively. These values are larger than those obtained for other multiferroic oxides, such as BiFeO_3_^30,31^, thus reflecting a strong electron-phonon interaction in YBaCuFeO_5_. As is evident from Fig. 4, the Bose–Einstein model reproduced the overall temperature dependence of the band gap in YBaCuFeO_5_ satisfactorily. However, the band gap deviated from the theoretical prediction at 455 and 175 K, which suggested spin–charge interactions. Similar coupling behavior has been observed in other multiferroic materials, such as BiFeO_3_^31,32^, *h*-LuFeO_3_^33^, and LuFe_2_O_4_^34^.

**Figure 5:**
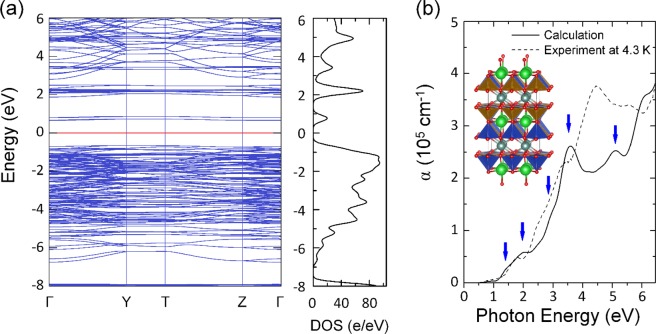
(**a**) Calculated electronic band structure, density of states (DOS), and (**b**) calculated and experimental optical absorption coefficient of YBaCuFeO_5_. The inset in (**b**) depicts the atomic structure of the simulation model with dark cyan, green, blue, brown, and red spheres representing the Y, Ba, Cu, Fe, and O atoms, respectively. The pyramids formed by oxygen atoms embracing Cu and Fe atoms are also highlighted.

To understand the nature of the observed absorption peaks in YBaCuFeO_5_, we calculated the electronic band structure, density of states (DOS), and optical absorption coefficient, and the results are presented in Fig. 5. In a previous report^13^, Morin *et al*. simulated YBaCuFeO_5_ models using DFT calculations with 10 arrangements of Cu and Fe atoms. However, optical absorption spectra and electronic band structure were not assessed in their work, and these properties are critical for analyzing the optical properties of YBaCuFeO_5_. We reinspected the crystal structure and electronic properties of the five models with the lowest energies from those in the study of Morin *et al*.^13^. In addition, we examined two models of slightly higher energies than the five previously mentioned models (see supplementary information). These two models were not investigated by Morin *et al*. We discovered that only one of the new models could reproduce the absorption spectrum presented in Fig. 2(b). The structure of the simulation model is depicted in Fig. 5(b).

The electronic band structure of YBaCuFeO_5_ (Fig. 5(a)) revealed that both the valence-band maximum and the conduction-band minimum were flat from the Γ to Y points of the Brillouin zone. The calculated direct band gap of YBaCuFeO_5_ was approximately 1.36 eV, which was consistent with the experimental results. Furthermore, the shape and peak positions of the calculated optical coefficient spectrum (Fig. 5(b)) exhibited favorable agreement with the experimental data. Electronic excitations occurred at approximately 1.45, 2.0, 2.9, 3.5, and 5.1 eV. According to the DOS analysis (see supplementary information), the observed excitation near 1.45 eV was due to transitions from the 3*d*_*xy*_ orbitals of Cu or Fe atoms to the 3*d*_*x*_^2^_−*y*_^2^ orbitals of Cu or Fe atoms. The absorption peak near 2.0 eV was attributed to transitions from the 3*d*_*xz/yz*_ orbitals of Cu or Fe atoms to the 3*d*_*x*_^2^_−*y*_^2^ orbitals of Cu or Fe atoms. These symmetry-forbidden *d-d* transitions were possible due to strong hybridization of Cu or Fe 3*d* and O 2*p* states. The observed 2.9-, 3.5-, and 5.1-eV absorption peaks were assigned to the charge–transfer excitations from the *2p* orbitals of O atoms to the 3*d* orbitals of Cu or Fe atoms.

### Vibrational properties

**Figure 6:**
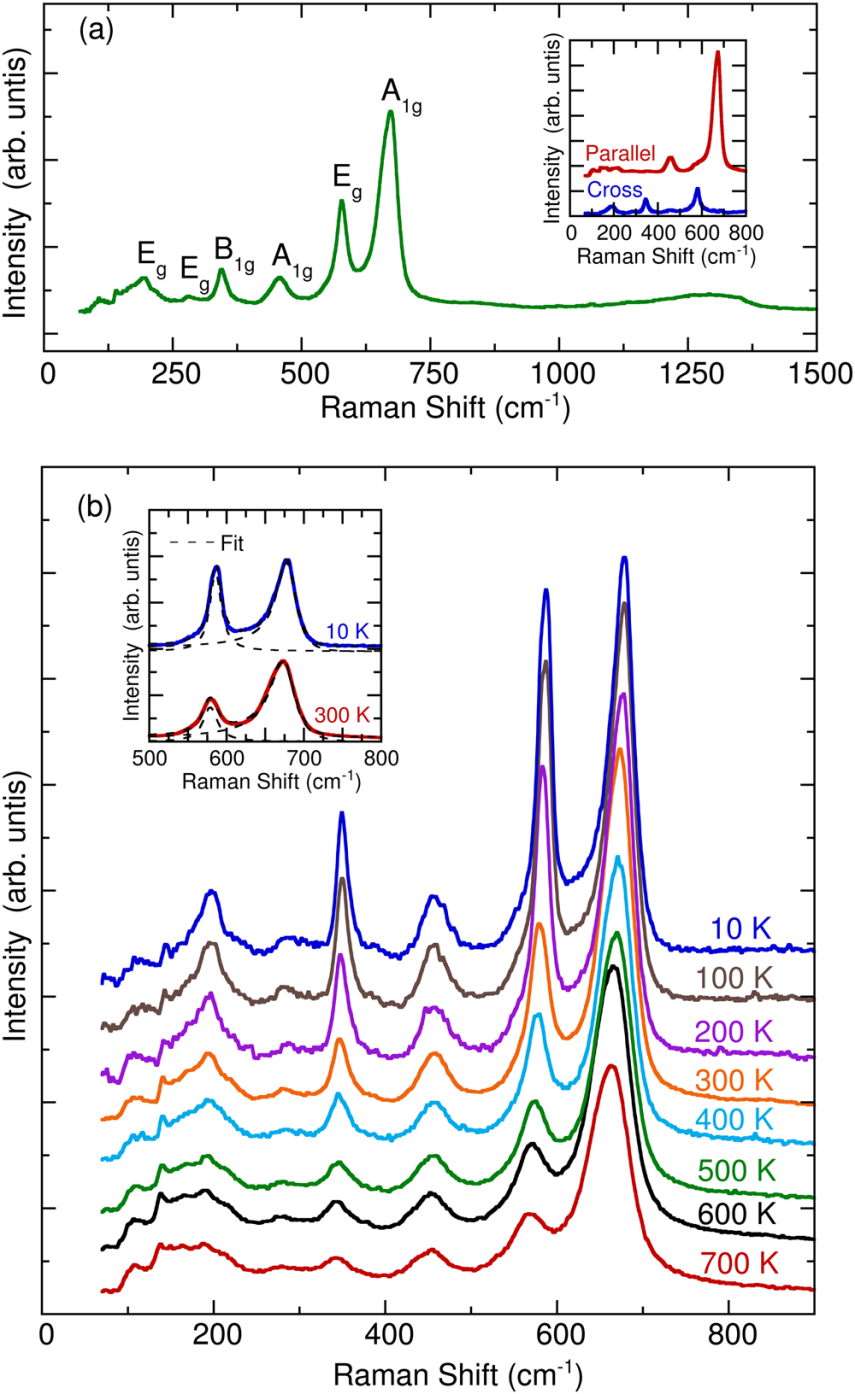
(**a**) Unpolarized Raman scattering spectrum of YBaCuFeO_5_ at room temperature. The inset illustrates the Raman scattering spectra in parallel and cross scattering geometries. (**b**) Temperature dependence of unpolarized Raman scattering spectra of YBaCuFeO_5_. The inset denotes the fitting results of spectra at 300 and 10 K using the Lorentzian and Fano models.

Figure 6(a) depicts the room-temperature Raman scattering spectrum of YBaCuFeO_5_. The spectrum comprised six first-order Raman phonon modes. We fitted these phonon peaks using a standard Lorentzian profile. According to factor group analysis, the structure of YBaCuFeO_5_ is tetragonal (space group P4/mmm) with one formula unit per primitive cell. The irreducible representation of the phonon modes at the center of the Brillouin zone is given by Γ = 2*A*_*1g*_ + *B*_*1g*_ + 3*E*_*g*_ + 5*A*_*2u*_ + 6*E*_*u*_ + *B*_*2u*_^3^. These modes are classified as Raman active (2*A*_*1g*_ + *B*_*1g*_ + 3*E*_*g*_), infrared active (4*A*_*2u*_ + 5*E*_*u*_), acoustic (*A*_*2u*_ + *E*_*u*_), and silent (*B*_*2u*_). The inset of Fig. 6(a) illustrates polarized Raman scattering spectra of parallel and cross measurements. The 456 and 675 cm^−1^ modes exhibited stronger intensities in the parallel configuration, indicating that they were of *A*_*1g*_ characters. By contrast, the 579 cm^−1^ mode in cross geometry was stronger than other phonon peaks, thus demonstrating *E*_*g*_ symmetry. The *E*_*g*_ mode involves in-plane O-Cu/Fe-O bending motions, whereas the *A*_*1g*_ mode results from out-of-plane Cu/Fe-O stretching motions^3^. Additionally, the low-intensity broad phonon mode observed at approximately 1267 cm^−1^ should be ascribed to multiphonon processes^35–37^. In the present study, the 675 cm^−1^ *A*_*1g*_ phonon mode exhibited an asymmetric line shape, indicating a strong interaction between the lattice vibrations and a magnetic continuum. As presented in the inset of Fig. 6(b), this mode was fitted using a standard Fano profile^38^ as follows: *I(ω*) = *I*_0_ (*ε* + *q*)^2^/(1 + *ε*^2^), where *ε* = (*ω* – *ω*_0_)/Γ; *ω*_0_ is the phonon frequency; Γ is the linewidth; and *q* is the asymmetry factor of the phonon mode.

**Figure 7:**
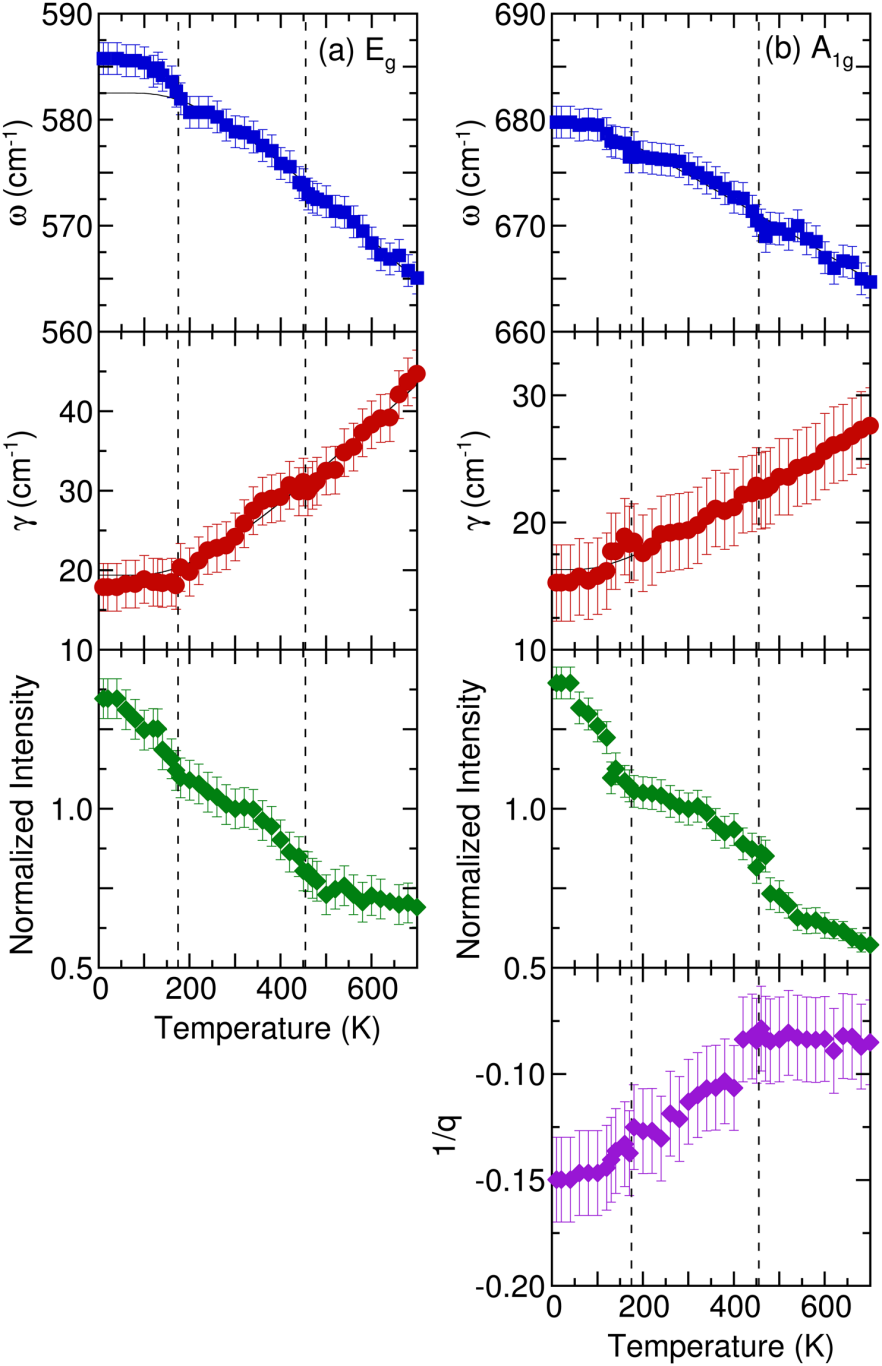
Temperature dependence of the frequency, linewidth, normalized intensity, and asymmetry factors of (**a**) *E*_*g*_ and (**b**) *A*_1*g*_ phonon modes of YBaCuFeO_5_. The thin solid lines indicate the results of the fitting from the anharmonic model. Vertical dashed lines denote transition temperatures.

Figure 6(b) indicates the temperature dependence of the Raman scattering spectra of YBaCuFeO_5_. With decreasing temperatures, the peak positions of all phonon modes shifted to higher frequencies, and the resonance linewidth narrowed. However, the phonon parameters of the 579 cm^−1^ *E*_*g*_ and 675 cm^−1^ *A*_1g_ modes behaved peculiarly at 455 and 175 K. Figure 7 further illustrates the frequency, linewidth, normalized intensity, and asymmetry factors of these two modes as functions of temperature. The frequencies and linewidths of the 579 cm^−1^ *E*_*g*_ and 675 cm^−1^ *A*_*1g*_ modes changed discontinuously at 455 and 175 K. Furthermore, the oscillator strengths of the two modes increased below 455 K. In a normal anharmonic solid, a nearly temperature-independent oscillator strength is expected, and at decreasing temperature, the phonon frequency should increase while linewidth decreases. Anharmonic interactions are relevant to high-order terms of atomic vibrations, which are beyond traditional harmonic terms. The temperature-dependent phonon frequency and linewidth can be written as^39^

4 $$\omega (T)={\omega }_{O}+A[1+\frac{2}{\exp (\hslash {\rm{\omega }}/{k}_{B}T)-1}],$$

and

5 $$\gamma (T)={\gamma }_{O}+B[1+\frac{2}{\exp (\hslash {\rm{\omega }}/{k}_{B}T)-1}],$$

where *ω*_*o*_ and *γ*_*o*_ are the intrinsic frequency of the optical phonon mode and the linewidth broadening that results from defects. Parameters *A* and *B* are the anharmonic coefficients, and $\frac{1}{[\exp (\hslash {\omega }_{o}/2{k}_{B}T)-1]}$ corresponds to the thermal population factor of acoustic modes. For analysis of anharmonic contributions to the 579 cm^−1^ *E*_*g*_ mode, the values of *ω*_*o*_ (≈582.5 cm^−1^), _*γo*_ (≈19 cm^−1^), *A* (≈−4.5), and *B* (≈1.8) were determined. For the 675 cm^−1^ *A*_*1g*_ mode, the values of *ω*_*o*_ (≈682.9 cm^−1^), _*γo*_ (≈16 cm^−1^), *A* (≈−4.9), and *B* (≈1.2) were determined. Parameter *A* was negative, indicating that with an increase in temperature, the peak shifted to a lower frequency because of anharmonic phonon decay. By contrast, parameter *B* was positive, reflecting linewidth narrowing with a decrease in the temperature. The thin solid lines in Fig. 7(a,b) represent theoretical predictions based on Eqs (4, 5). The 675 cm^−1^ *A*_*1g*_ mode exhibited a slight deviation from the usual anharmonic contribution to the temperature dependence of the phonon frequency and linewidth through the 455 and 175 K antiferromagnetic ordering transitions. By contrast, the 579 cm^−1^ *E*_*g*_ mode hardened significantly below 175 K. Because the YBaCuFeO_5_ exhibited no drastic temperature dependence in terms of crystal structure and lattice parameters, the phonon anomalies below the antiferromagnetic ordering temperature were attributed to spin–phonon interactions. Similar magnetoelastic coupling has been detected in other multiferroic materials, such as HoMnO_3_^40^, BiFeO_3_^41^_,_ and Y_2_CoMnO_6_^42^.

As indicated in Fig. 7(a), the frequency of the 579 cm^−1^ *E*_*g*_ mode deviated considerably from the theoretical predictions to below the antiferromagnetic phase transition temperature of 175 K. This effect was attributed to the renormalization of the in-plane *E*_*g*_ phonon induced by magnetic ordering, usually understood as a sign of coupling between the lattice and spin degrees of freedom. The spin–phonon coupling constant can be calculated from frequency–shift data using the expression^43^

6 $${({\rm{\Delta }}\omega )}_{SP}=\frac{{\lambda }_{eff}}{{\omega }_{{0}}}{[\frac{{M}_{sub}(T)}{4{\mu }_{B}}]}^{2},$$

where (Δ*ω*)_*SP*_ is the difference between the phonon frequency values at 10 K from the theoretical prediction and experimental data; *ω*_o_ is the value from Eq. (4); *M*_*sub*_*(T)* is the sublattice magnetic susceptibility with the external magnetic field along the *ab* plane; and *λ*_*eff*_ is the spin–phonon coupling constant. In the present study, we used the magnetic susceptibility with the external magnetic field along the *ab* plane and estimated the spin–phonon coupling constant to be 15.7 mRy/Å^2^. The magnitude of this value was comparable to those obtained for other multiferroic manganites^44–46^.

## Summary

We employed both spectroscopic ellipsometry and Raman scattering spectroscopy to investigate the electronic structure and lattice dynamics of YBaCuFeO_5_ single crystals. We also characterized the optical transitions by comparing the experimental data and the results of first-principles calculations. The optical absorption spectrum of YBaCuFeO_5_ at room temperature revealed Cu/Fe *d* to *d* on-site transitions at approximately 1.69 and 2.47 eV and O 2*p* to Cu/Fe 3*d* charge–transfer transitions at approximately 3.16, 4.26, and 5.54 eV. YBaCuFeO_5_ exhibited a direct band gap of 1.41 ± 0.01 eV at 300 K. The band gap presented anomalies through antiferromagnetic phase transition temperatures at 455 and 175 K. Moreover, the temperature dependence of in-plane *E*_*g*_ phonon mode exhibited a hardening below 175 K. The spin–phonon coupling constant was estimated to be 15.7 mRy/Å^2^. These results confirmed a strong coupling of spin, charge, and lattice degrees of freedom in YBaCuFeO_5_.

## Supplementary information

Dataset 1
