# Supplementary material for: Spin-charge-lattice coupling in YBaCuFeO5: Optical properties and first-principles calculations
Source: Sci Rep. 2019 Mar 1;9:3223. doi: 10.1038/s41598-019-39031-6 (PMC6397168; doi:10.1038/s41598-019-39031-6)
Supplement: Supplementary file 1 — Dataset 1 [file 41598_2019_39031_MOESM1_ESM.pdf]

Supplementary information

Spin-charge-lattice coupling in $YBaCuFeO_5$: Optical properties and first-principles calculations

H. W. Chen[1], Y.-W. Chen[2], J.-L. Kuo[2], Y. C. Lai[3,4], F. C. Chou[3], C. H. Du[4], and H. L. Liu[1,*]

[1]Department of Physics, National Taiwan Normal University, Taipei 11677, Taiwan

[2]Institute of Atomic and Molecular Sciences, Academia Sinica, Taipei 10617, Taiwan

[3]Center for Condensed Matter Sciences, National Taiwan University, Taipei 10617, Taiwan

[4]Department of Physics, Tamkang University, Tamsui, New Taipei City 25137, Taiwan

*Corresponding author: hliu@ntnu.edu.tw

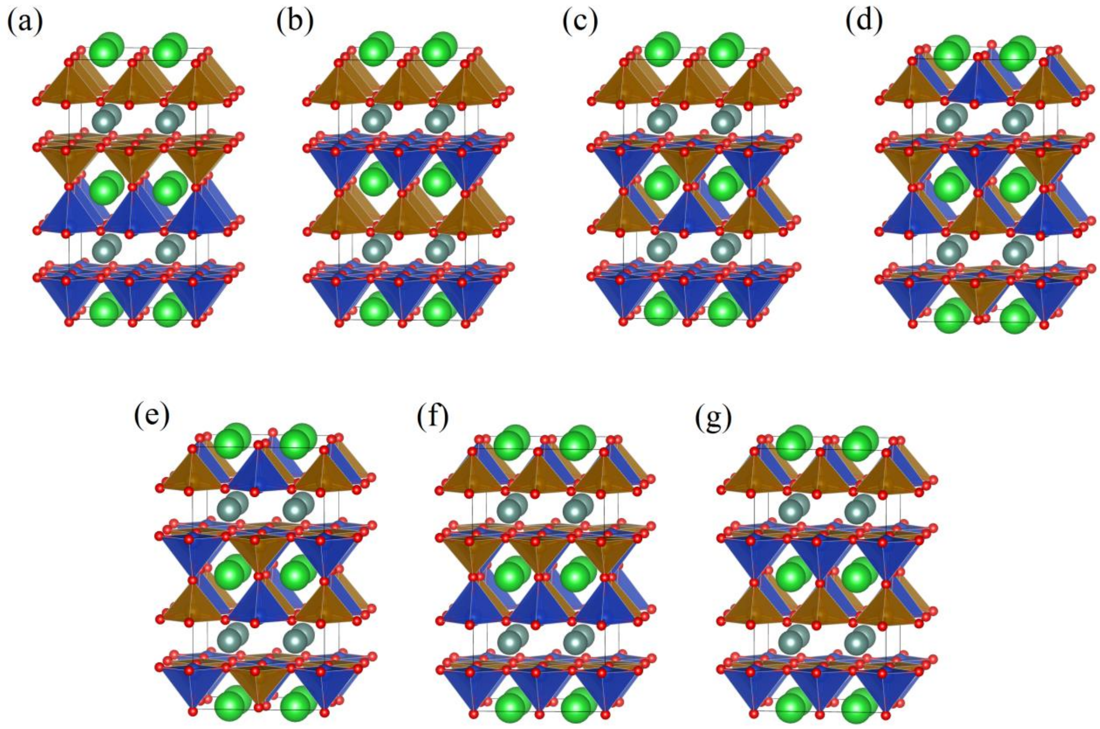

Figure S1: The structures of seven DFT models. The dark cyan, green, blue, brown, and red spheres represent the Y, Ba, Cu, Fe, and O atoms, respectively. The pyramids formed by oxygen atoms embracing Cu and Fe atoms are also highlighted. The models (a) to (e) are the same five structures (a) to (e) with lowest energies in M. Morin *et al.*, Phys. Rev. B **91**, 064408 (2015). The (f) and (g) models are new models in this work.

Table S1. The lattice constants and relative energies (one formula cell, $YBaCuFeO_5$) of seven DFT models as shown in Figure S1.

Structure	(a)	(b)	(c)	(d)	(e)	(f)	(g)
a (Å)	3.883	3.879	3.892	3.902	3.903	3.917	3.926
b (Å)	3.883	3.879	3.892	3.902	3.903	3.886	3.877
c (Å)	7.928	7.968	7.859	7.776	7.757	7.786	7.788
Energy (eV)	0.015	0	0.092	0.177	0.169	0.130	0.135

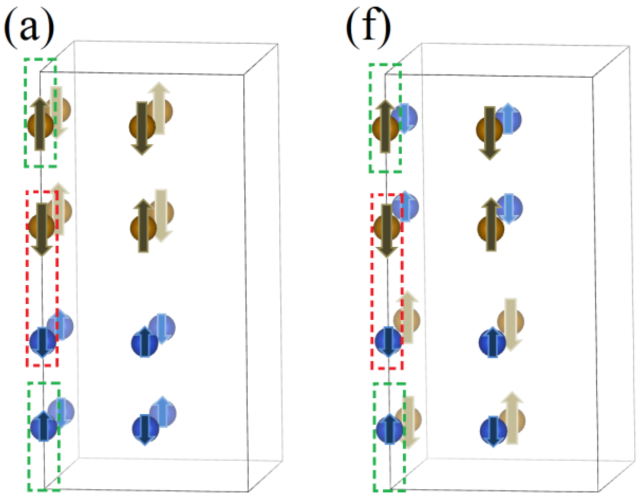

Figure S2: The G-type AFM arrangement of spin moments on the Fe (brown color) and Cu (blue color) atoms in (a) and (f) DFT models as shown in Figure S1. We count the spins on Fe and Cu atom embraced in the dashed red box in (a) or (f) as one spin moment together (ie. the Fe, Cu atom pair embraced in the top and bottom pyramids connected by one oxygen atom); the spins in the dashed green boxes should be counted as another one spin as well. In total, there are eight paired spin moments forming G-type AFM arrangement.

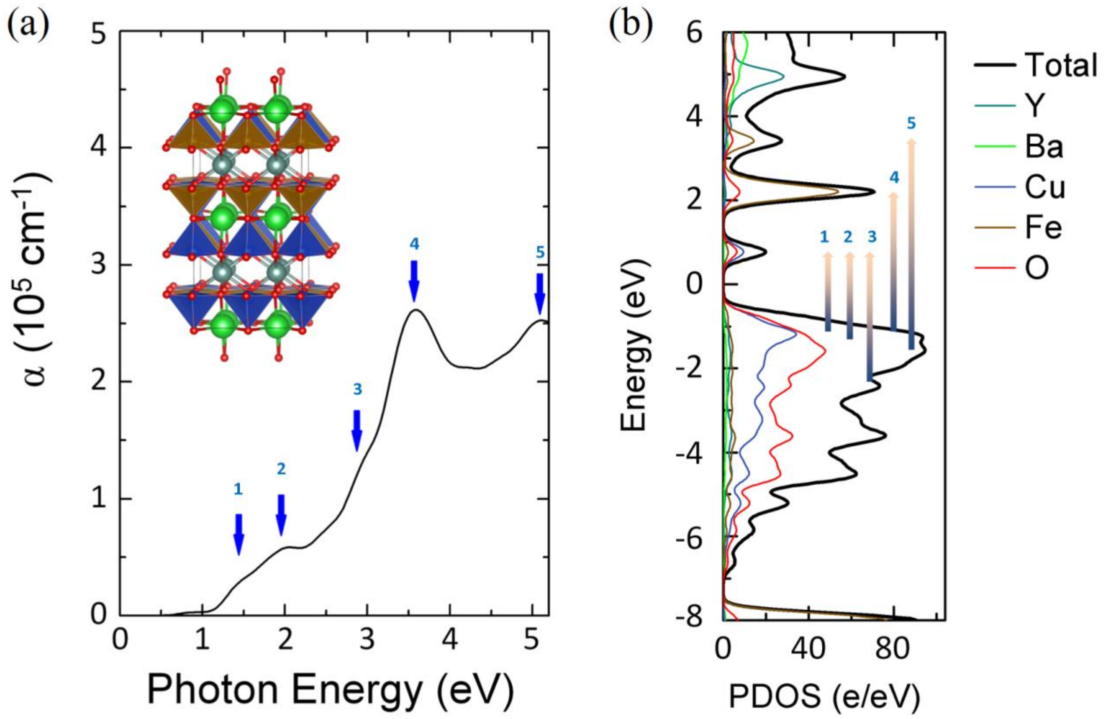

Figure S3: (a) optical absorption coefficient and (b) the electron transition assignment from PDOS (projected density of states) analysis of $YBaCuFeO_5$ structure (f) in Figure S1. The number 1 to 5 absorption peaks in (a) are assigned to the electron transition assignment number 1 to 5 in (b).

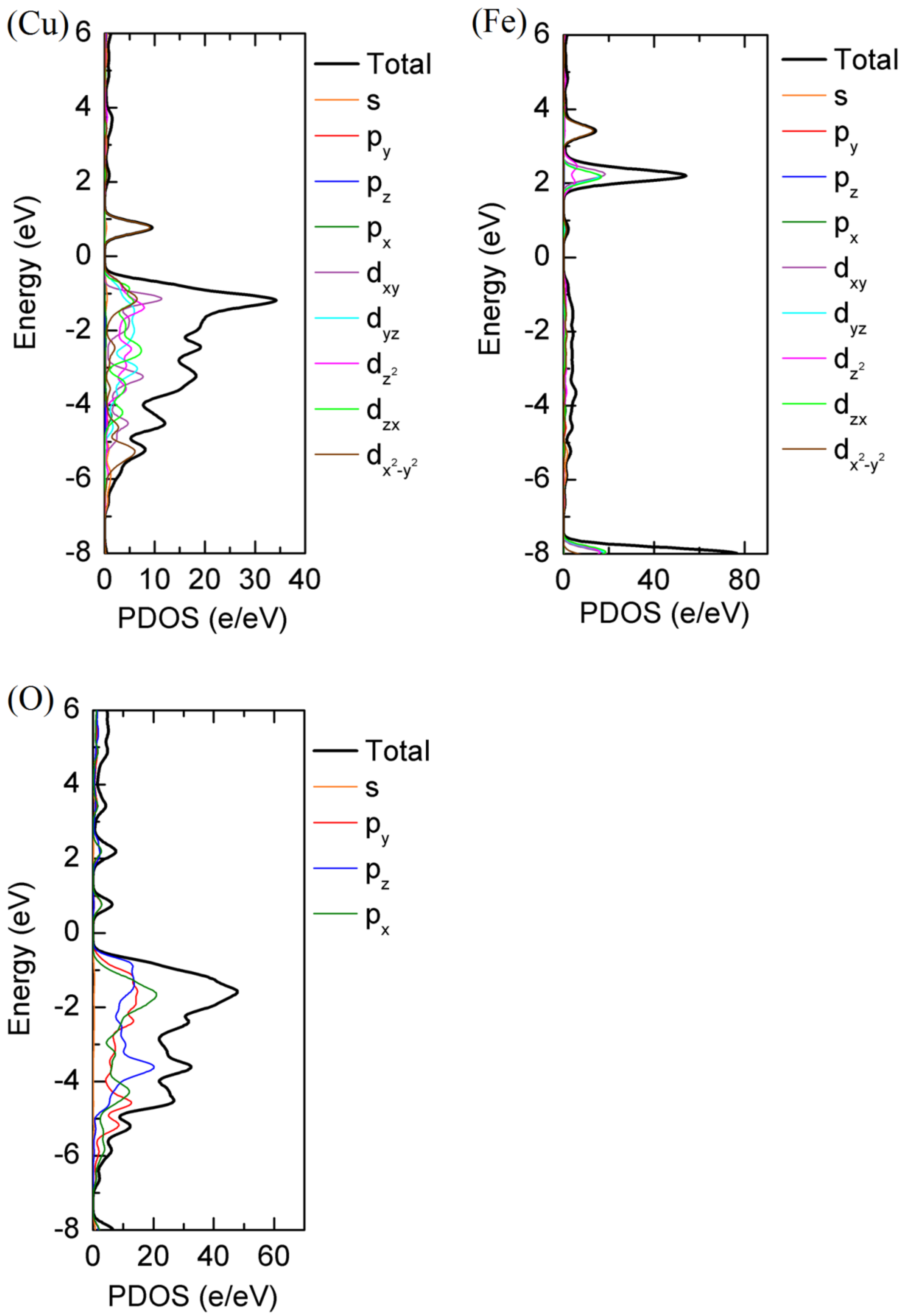

Figure S4: the orbital decompositions of PDOS for Cu, Fe, and O atoms.
